# Attending an Historically Black College or University and all-cause mortality in US Black adults

**DOI:** 10.1093/aje/kwag141

**Published:** 2026-06-19

**Authors:** Marilyn D. Thomas, Carol Wei, Min Hee Kim, Jennifer J. Manly, Suzanne E. Judd, Justin S. White, Virginia J. Howard, Christina Mangurian, Rita Hamad, M. Maria Glymour

**Affiliations:** 1 Department of Medicine, Division of General Internal Medicine, University of California San Francisco; 2 Department of Epidemiology and Biostatistics, University of California San Francisco; 3 Philip R. Lee Institute for Health Policy Studies, University of California San Francisco; 4 Center for Health Services Research, Institute for Health, Health Care Policy and Aging, School of Nursing, Rutgers University, New Brunswick, NJ.; 5 Department of Psychiatry and Behavioral Sciences, University of California San Francisco; 6 Department of Health Behavior, School of Public Health, University of Alabama at Birmingham; 7 Columbia University Irving Medical Center, New York, NY; 8 Department of Health Law, Policy, & Management, Boston University School of Public Health; 9 Department of Epidemiology, School of Public Health, University of Alabama at Birmingham; 10 Department of Social and Behavioral Sciences, Harvard T.H. Chan School of Public Health; 11 Department of Epidemiology, Boston University School of Public Health

## Abstract

Recent studies suggest that attending a Historically Black College or University (HBCU) versus attending a Predominantly White Institution (PWI) may improve later-life health outcomes for Black adults. No studies have evaluated differences in all-cause mortality. The REGARDS prospective study recruited US adults aged 45 and older during 2003–2007. Black college-goers who attended high school in a state with an HBCU (N=1,960) were classified as ever attending an HBCU or only a PWI (reference). Cox proportional hazards models estimated adjusted mortality ratios (2012–2023) and effect modification by being college-aged before 1955 (during legal racial segregation), 1955–1964 (before the Civil Rights Act), or after 1964. Mean age was 62 years (SD±8.2) and 35% attended an HBCU. HBCU versus PWI attendees had negligibly lower all-cause mortality and estimates were imprecise (HR 0.99; CI 0.88, 1.12). Although confidence intervals were wide, those college-aged during 1955–1964 had lower mortality (HR 0.87; CI 0.71, 1.06) but elevated mortality for those college-aged before 1955 (HR 1.03; CI 0.85, 1.24) and after 1964 (HR 1.46; CI 0.91, 2.33). Inconsistent with prior work, we found little evidence that HBCU attendance was associated with lower mortality yet could not rule out substantial protective or harmful effect modification by college-aged years.

## INTRODUCTION

In the United States (US), Black-White inequities in mortality are a pressing public health problem.^1–3^ Although life expectancy has steadily increased from 1999–2015 for both Black and White Americans, Black-White disparities in the leading causes of death persist across all age groups under 65 years.^4^ A recent systematic analysis reported a national age-adjusted average all-cause mortality rate (per 100,000) of 954 for Black and 803 for White Americans.^2^

Racial differences in mortality are consistently driven by social determinants in health.^3–8^ Lower educational attainment and income, poor health behaviors, and allostatic load are key drivers of higher all-cause mortality for Black compared to White adults.^5–10^ Geronimus’s *weathering hypothesis*^11^ posits that Black Americans experience accelerating aging from early physiologic deterioration due to chronic exposure to psychosocial stressors (i.e., higher allostatic load^12–14^), which has been linked to racial disparities in mortality.^9,15^ A major gap in the literature is our lack of understanding about whether culturally-specific social factors mitigate mortality risk in Black adults.

Higher education is a principal social determinant of reduced risk of accelerated aging and mortality.^16–18^ People with higher education have greater access to health-promoting resources that contribute to increased longevity. However, structural racism is implicated as a fundamental cause of Black–White inequities in mortality^19,20^ even among those with a college education.^21^ For instance, the protective association between higher education and lower risk of early all-cause mortality was attenuated for Black adults residing in states with a smaller Black–White ratio of citizens with a bachelor’s degree, an indicator of higher structural racism.^20^ Moreover, higher education has been shown to buffer the adverse link between experiences of racism and accelerated aging in Black adults.^22,23^ This evidence points to a need to examine educational sources of resilience that may be differentially protective for Black Americans compared to the general population. For example, attendance at Historically Black Colleges and Universities (HBCUs) may offer important advantages to Black students.

After the Civil War, HBCUs provided access to higher education for Black Americans who were barred from White colleges.^24^ Presently, over 3 in 4 HBCU students are Black^25^ largely because HBCUs encourage Black pride and tradition, advance high-quality education with prominent Black scholars, reduce exposure to racial stressors, and increase community building.^26–28^

Compared to Black students attending Predominantly White Institutions (PWIs), those attending HBCUs report better academic achievements, increased social involvement, and robust alumni networks to cope with stressors.^29–32^ Black HBCU versus PWI attendees also have greater occupational goals and earn higher salaries^31–33^ both of which are associated with healthier behaviors and greater access to health-promoting resources. Hence, HBCU attendance may be a culturally-relevant environmental exposure that has a distinct influence on health over the life course of Black adults, and ultimately risk of mortality.

A growing body of evidence suggests that attending an HBCU versus a PWI improves Black health, yet few studies have evaluated the influence of HBCU attendance on physical health outcomes. The majority of health studies has focused on HBCU exposure and behaviors (e.g., substance use, sexual activity), coping strategies (e.g., Superwoman Schema, John Henryism), and mental wellbeing (e.g., depression, distress).^29,30,34–43^ Two studies have reported on physical health in Black adults by midlife: HBCU (vs. PWI) attendance was associated with a reduced risk of metabolic syndrome^44^ and improved cognition.^45,46^ To date, the impact of HBCU attendance on risk of mortality is not known.

In this national cohort study of US Black adults who attended college, we compared the probability of survival by mid-to-later life between those attending an HBCU versus a PWI. Because participants were differentially exposed to racialized education policies (i.e., 1954 *Brown vs. Board of Education* ruling and the 1964 Civil Rights),^24^ secondary analyses included effect modification by whether the respondent was nearing college-age (17 years) before 1955 (during legal racial segregation in school), 1955–1964 (before barring racial discrimination in education), or after 1964. We hypothesized that compared to PWI attendance, HBCU attendance would be linked to lower all-cause mortality for Black adults, and that the magnitude of this association would be lowest for HBCU attendees during racial segregation and highest after racial discrimination was barred.

## METHODS

### Study Participants

The REasons for Geographic and Racial Differences in Stroke (REGARDS) study, a prospective cohort study, recruited Black and White US adults aged 45 and older during 2003–2007.

REGARDS oversampled Black residents from the southeastern “Stroke Belt” (56%), a group of eight Southern states defined by excess stroke mortality.^47^ Detailed methods are described elsewhere.^48^ In brief, baseline demographic, medical, and lifestyle information were collected via a computer-assisted telephone interview and trained interviewers. Every 6 months, interviews collect information for clinical events, including death. In 2012, data collection was initiated on childhood and family life factors as well as college attendance information. Informed consent was obtained verbally and in writing. All REGARDS study methods were approved by participating institutional review boards.

**Figure 1.**
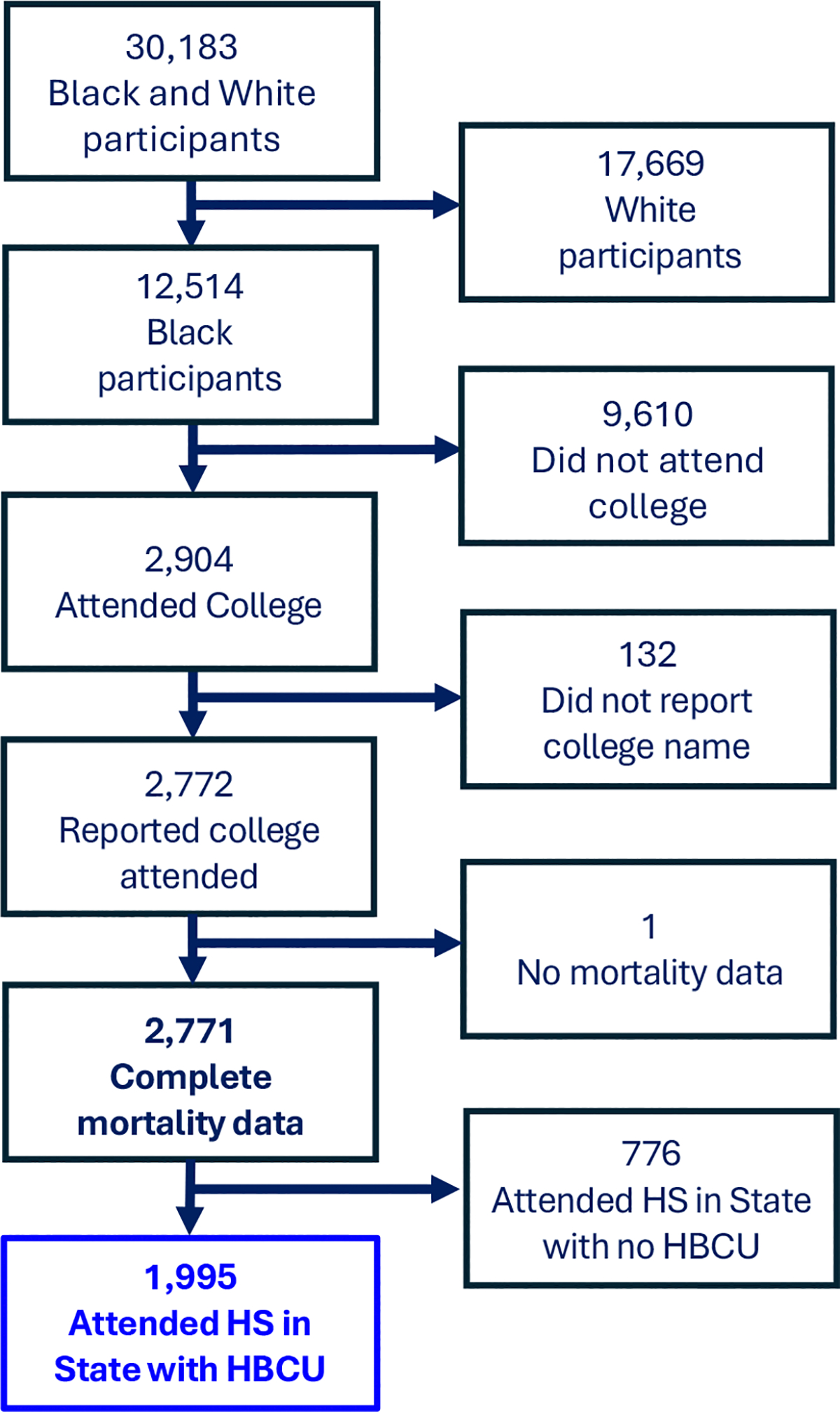
Process of sample selection among REasons for Geographic and Racial Differences in Stroke (REGARDS) study participants. Abbreviation: HBCU = historically Black college or university

As shown in Figure 1, among 12,514 Black participants, 2,904 (23%) attended college. Among those reporting the name of the college attended (N=2,772; 96%), 2,724 (98%) had complete mortality data. We restricted our primary analytic sample to the 1,960 Black participants who attended a high school in a state with an HBCU (19 states), including DC, and would thereby have similar probabilities of HBCU attendance. To assess the robustness of our methodology, we conducted secondary analyses to include (1) Black participants in all states and (2) an HBCU by birth cohort interaction term to evaluate potential effect modification by whether the respondent was nearing college-age before 1955, 1955–1964, or after 1964.

### Exposure Measure

In the US, there are 107 established HBCUs.^25,49^ A comprehensive cleaning of all college names was performed (e.g., spell checks, abbreviations, etc.). HBCU location by county and state was confirmed using participant residential history. A dichotomous measure indicated whether the participant had ever attended an HBCU (0=PWI, 1=HBCU).

### Outcome Measure

When a death occurred, next of kin or proxies were interviewed to obtain the date and circumstances surrounding death. Medical records for hospitalizations or emergency department visits in the months preceding death, along with death certificates including cause(s) of death, autopsy reports, and data from the National Death Index were collected by REGARDS for central adjudication. Each participant’s death was linked to the National Death Index within one year of death by REGARDS through 2023. A dichotomous all-cause death indicator was derived from each participant’s survival status using survival through the 2023 follow-up.

### Covariates

A directed acyclic graph was used to represent hypothesized causal pathways and represent most concerning confounding early-life domains (Figure S1): socioeconomic status, academics, health, social support, and state-level factors. Because each domain had multiple, often highly correlated, indicators, we used a data-driven approach to select the most pertinent measures within each domain. For each pre-specified domain, we performed bivariable analyses (i.e., chi-square tests) to assess the strength of associations between independent and outcome variables available in REGARDS at alpha=0.10 and selected the strongest predictors (Tables S1 & S2). Final models were adjusted for sex (female, male), community size at birth (city/large town, rural/small town), college-aged years (<1955, 1955–1964, >1964), and, retrospectively reported childhood factors, including mother/female caregiver’s education (college/post high school, less than high school/unknown), general health (excellent/very good, good/fair/poor), encouragement to succeed in school (all/most/some of the time, little/none/unknown), death of a parent (no/unknown, yes), and state of high school attended.

### Statistical Analysis

Analyses were conducted using STATA 18.0 (StataCorp, College Station, TX). Frequencies and central tendencies described participant characteristics. Chi-square and t-tests were used to evaluate differences between HBCU and PWI attendees. Multiple imputation by chained equations (*m*=50) was used to handle missing data (<16%) to retain the entire sample and improve estimate precision.^50,51^ Postestimation imputation checks were performed using *vartable* and *nocitable* commands.

Childhood and college attendance data were collected in 2012, hence years of follow-up from 2012 to 2023 was used as the timescale (i.e., year at death was the dependent variable). Cox proportional hazards regression models were estimated to compare the mortality risk for HBCU versus PWI attendees using the *stcox* command, adjusting for participant characteristics likely to be confounders (listed above) and state fixed effects using the *strata* option. We clustered standard errors by participants’ high school state to account for correlated outcomes resulting from attending high school in the same state using *vce(cluster)*. Kaplan Meier curves illustrating the probability of survival over the study period were generated using *sts graph*. The proportional hazards assumption was evaluated by a global test of Schoenfeld residuals^52^ using *estat phtest* and by plots comparing observed and predicted curves using *stcoxkm*.

## RESULTS

### Participant Characteristics

The mean baseline age of participants was 62 years (SD±8.2) and 35% (694/1,960) attended an HBCU (Table 1). Compared to PWI attendees, HBCU attendees were slightly older (aged 63 vs. 61). During childhood, HBCU attendees were more likely to have a mother/female caregiver with a college education (20% vs. 9%), encouragement to succeed in school (73% vs. 57%), and experience no death of a parent (65% vs. 57%). On average, participants had 8.9 years of follow-up interviews (range 0.1–12.2) and 26% did not survive: 28% for HBCU attendees (193/694) and 25% for PWI attendees (318/1,266). There was no difference in mean age at death for HBCU and PWI attendees (p=0.67): 81.7 years (SD±9.1). The distribution of participant characteristics stratified by HBCU attendance and years when college-aged are presented in Table S3.

### Survival Analysis

After accounting for early-life factors, we found little evidence that HBCU attendance was associated with all-cause mortality in the full cohort (HR 0.99, 95% confidence interval [CI] 0.88, 1.12) (Table 2). The hazard ratio associated with HBCU attendance for participants attending high school in a state with an HBCU was comparable to the estimate for participants attending high school in all states (HR 0.98, CI 0.89, 1.07). Kaplan Meier curves are illustrated in Figure S2.

Postestimation assessments showed that performing 50 imputations resulted in high relative efficiency (>99%) with negligible increases in variance due to the replacement of missing values. Both visual examination of survival curves and tests of Schoenfeld residuals suggested that the proportional hazards assumption was reasonable (Figure S3).

### Effect Modification

**Figure 2.**
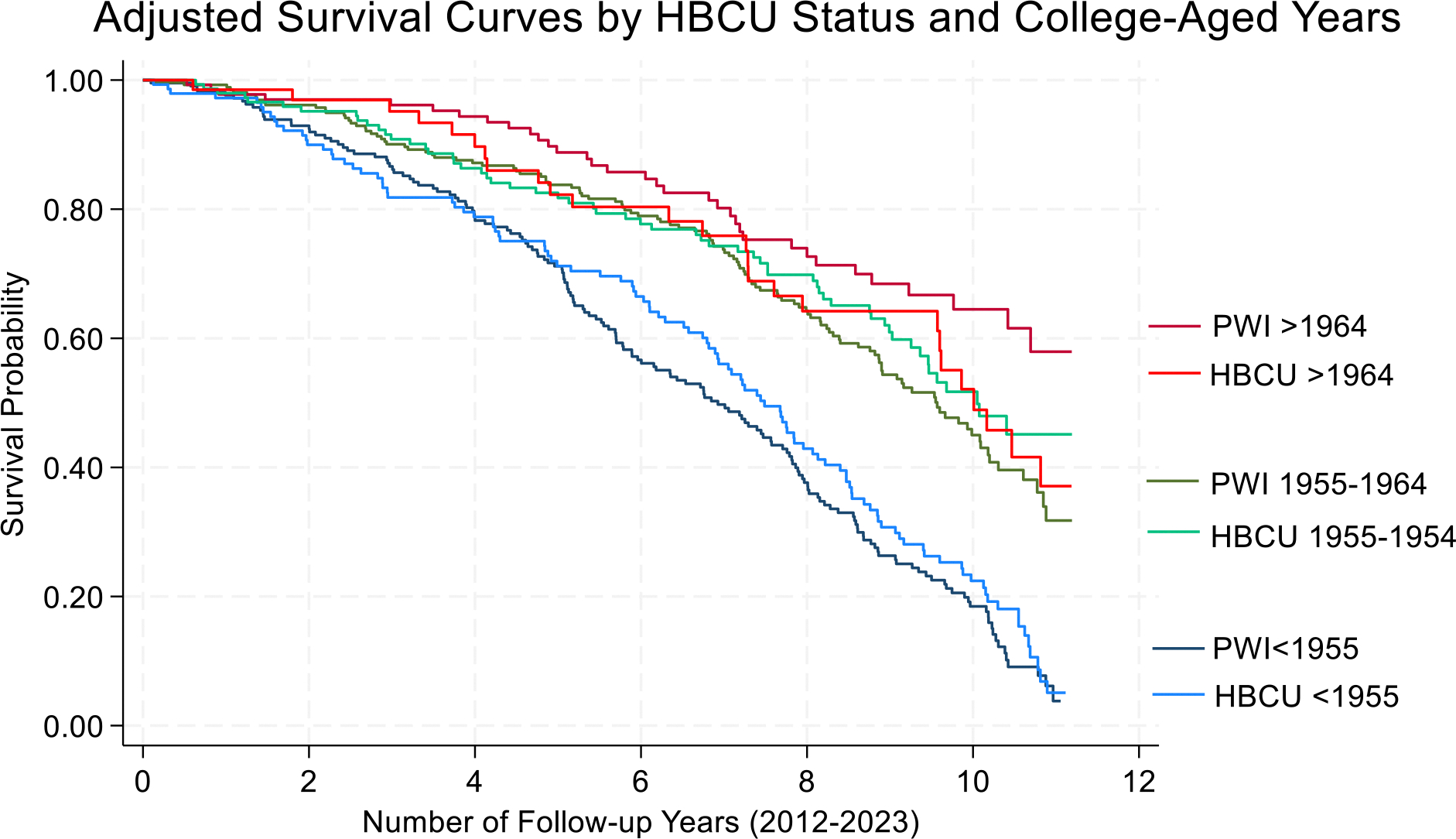
Kaplan Meier survival curves among Black adults in the REasons for Geographic and Racial Differences in Stroke (REGARDS) study who attended high school in an state with an historically Black college or university (HBCU) and attended an HBCU versus a predominantly White institution (PWI), stratified by years when college-aged prior to the *Brown vs Board of Education* court decision ending legal racial segregation of schools (<1955), prior to the Civil Rights Act (CRA) prohibiting racial discrimination in education (1955–1964), or after the adoption of the CRA (>1964).

HBCU attendance interacted with indicators for the years when the participant was college-aged (p=0.001) hence estimates are reported stratified by years when college-aged (Table 2). Confidence intervals were wide; however, the magnitude and direction of each estimate varied substantially. As shown in Figure 2, compared to PWI attendees, HBCU attendees who were college-aged during 1955–1964 had lower mortality (HR 0.87, CI 0.71, 1.06) while mortality was slightly higher for those college-aged before 1955 (HR 1.03, CI 0.85, 1.24) and notably higher for those college-aged after 1964 (HR 1.46, CI 0.91, 2.33).

## DISCUSSION

This is the first study to examine the effects of HBCU versus PWI attendance on mortality in US Black adults. Among 1,960 Black college-goers from states with an HBCU (mean base age 62), 35% attended an HBCU. As hypothesized, all-cause mortality was lower for HBCU versus PWI attendees however effect estimates were imprecise. This finding was consistent among participants in all states. As hypothesized, there were differences in this association across subgroups who were college-aged during legal racial segregation, before the Civil Rights Act, and after the Civil Rights Act, yet findings were not fully in expected direction. Because confidence intervals were wide, we could not rule out substantial protective or harmful effects, suggesting that further investigation is needed.

Notably, study participants may not have been old enough and/or followed long enough to reach meaningful differences in mortality. Unlike chronic disease development, mortality is a long-term outcome that occurs more frequently in late-life. As such, adult mortality risk is often studied in elderly populations (> 65 years) and/or over long follow-up periods (> 20 years).^18^ The current study participant mean age was nonelderly (62 years) with an average follow-up of 9 years. Instead, HBCU attendance may be a stronger predictor of common risk factors of mortality (e.g., allostatic load, hypertension, dementia^53–56^), in this non-elderly cohort.

We found evidence that the impact of HBCU attendance varied by years when participants were college-aged. Although many factors changed across birth cohorts, the changes in nationwide race-based education policies seem especially pertinent. We hypothesized that as racial equity in access to higher education increased, the probability of mortality risk would decrease for HBCU versus PWI attendees. The lower mortality among those who were college-aged after the end of legal racial segregation aligned with this hypothesis and with evidence demonstrating the benefits of desegregation on Black health.^57,58^ However, the higher probability of mortality for HBCU attendees after the Civil Rights Act passed was unexpected. This finding may be explained in part by factors related to the sudden change in college choice and selectivity for talented and affluent Black enrollees.^24,59^ The 1964 Civil Rights Act created financial incentives for PWIs to recruit Black students. Thus, the enrollment of high-achieving and higher socioeconomic status Black students reportedly increased at top-tier PWIs (e.g., Harvard, Yale) and decreased at HBCUs during the late 1960s and then stabilized by the 1980s.^24,59^ Although we accounted for some of these early-life predictors in our study, we were not able to account for factors that may have influenced the shift in college selection (e.g., private high school attendance, college admission test scores). Larger nationally representative prospective studies are needed for a conclusive understanding.

Future mediation analyses using nationally representative longitudinal data studies would also help elucidate biopsychosocial and economic pathways linking HBCU attendance to all-cause mortality. Specifically, long-term HBCU benefits are likely mediated through degree attainment and socioeconomic position, although this was not the focus of our study. Black HBCU (vs. PWI) attendees have better degree achievements and higher incomes,^31,32^ two socioeconomic indicators associated with lower rates of mortality for Black adults.^3,6–8^ HBCUs also enhance the resilience of Black communities through increased political empowerment, economic stability, and cultural capital^60,61^ such that ecological studies may provide key insights. Plausibly, HBCU exposure may reduce mortality risk for Black adults through socially inclusive environments, reduced exposure to race-related stressors, engagement in healthier behaviors, and increased resources to cope with stressors that contribute to the development of chronic disease.

This study’s strengths include the use of a unique data source with retrospective childhood information and residential history including individuals from across the US with some overrepresentation in the Southern states. Internal validity was improved by accounting for childhood factors to help minimize selection bias from left truncation or loss to follow-up yet recall bias is possible. Moreover, HBCU attendance is a novel feature of early-life Black educational experiences that may provide a source of resilience against a disproportionate risk of later-life mortality. However, limitations include a non-probability sample, such that findings may not be generalizable. Although our inferences rely on longitudinal data with unusually strong covariate control, causal inferences still rest on the assumption that we have adequately captured all factors that influence both HBCU attendance and mortality. Finally, HBCU attendance does not directly capture several institutional characteristics that may be culturally-relevant for long-term Black health (e.g., Black faculty, acculturation, leadership, fraternities) which may occur to varying degrees in both HBCUs and PWIs.

## CONCLUSION

In a national cohort of Black college-goers, we found little evidence that HBCU versus PWI attendance was associated with all-cause mortality by mid-to-later life. We found differences in this association by the years when participants were college-aged, which may reflect effect modification by racialized education policies which changed over time. Because confidence intervals were wide, we could not rule out moderate protective or harmful effects or fully explore heterogeneity. Larger nationally representative studies are needed for a conclusive understanding of the impact of HBCU attendence on Black mortality.

## Supplementary Material

supplemental

## Figures and Tables

**Table 1.** Characteristics of REGARDS participants who attended high school in a state with an HBCU by type of college attended

Variables	HBCU (n=694)	PWI (n=1,266)	Total (N=1,960)	p-value

	n (%)	n (%)	n (%)	
**Baseline age** (mean, SD)	62.7 (8.0)	61.3 (8.2)	61.8 (8.2)	< 0.001
**Sex**				
Male	231(33.3)	411(32.5)	642 (32.8)	0.711
Female	463 (66.7)	855 (67.5)	1,318 (67.2)	
**Years when college-aged**				
Pre-Brown (< 1955)	205 (29.5)	312 (24.6)	517 (26.4)	0.008
Post-Brown/Pre-CRA (1955–64)	318 (45.8)	568 (44.9)	886 (45.2)	
Post-CRA (> 1964)	171 (24.6)	386 (30.5)	557 (28.4)	
**Community size at birth**				
City/Large Town	246 (35.5)	445 (35.2)	691 (35.3)	0.945
Rural/Small Town	421 (60.7)	775 (61.2)	1,196 (61.0)	
Missing	27 (3.9)	46 (3.6)	73 (3.7)	
**Mother's/Caregiver's education**				
College	137 (19.7)	108 (8.5)	245 (12.5)	< 0.001
Post HS Vocational/Professional	44 (6.3)	74 (5.9)	118 (6.0)	
HS/GED	100 (14.4)	185 (14.6)	285 (14.5)	
<HS/None/Unknown	406 (58.5)	880 (69.5)	1,286 (65.6)	
Missing	7 (1.0)	19 (1.5)	26 (1.3)	
**Childhood encouragement to succeed in school**				
All the time	504 (72.6)	718 (56.7)	1,222 (62.4)	< 0.001
Most of the time	90 (13.0)	204 (16.1)	294 (15.0)	
Some of the time	35 (5.0)	130 (10.3)	165 (8.4)	
Little of time	21 (3.0)	90 (7.1)	111 (5.7)	
None of the time	10 (1.4)	67 (5.3)	77 (3.9)	
Missing	34 (4.9)	57 (4.5)	91 (4.6)	
**Childhood general health**				
Excellent/Very Good	487 (70.2)	861 (68.0)	1,348 (68.8)	0.251
Good/Fair/Poor	185 (26.7)	375 (29.6)	560 (28.6)	
Missing	22 (3.2)	30 (2.4)	52 (2.7)	
**Death of a parent in childhood**				
No/Unknown	452 (65.1)	723 (57.1)	1,175 (60.0)	0.001
Yes	160 (23.0)	323 (25.5)	483 (24.6)	
Missing	82 (11.8)	220 (17.4)	302 (15.4)	
**Follow-up Years** (mean, SD)	8.9 (3.6)	9.0 (3.6)	8.9 (3.6)	0.507
**Survival to 2023**				
No	193 (27.8)	318 (25.1)	511 (26.1)	0.194
Yes	501 (72.2)	948 (75.9)	1,449 (73.9)	
**Age at death** (mean, SD)	81.9 (9.4)	81.6 (8.9)	81.7 (9.1)X	0.671

Abbreviations: REGARDS = REasons for Geographic and Racial Differences in Stroke, PWI = predominantly White institution, HBCU = historically Black college or university, CRA = Civil Rights Act, HS = high school, GED = general education diploma, SD = standard deviation

**Table 2.** Adjusted hazard ratios estimating the probability of all-cause mortality of HBCU versus PWI attendees among Black adults in REGARDS and estimates stratified by college-aged exposure to legal racial segregation (Pre-*Brown*) and Civil Rights Act (CRA)

Outcome	N	HR	95% CI	p-value

**Primary Analysis**				
**HBCU States**	1960	0.99	(0.88, 1.12)	0.896
**Secondary Analyses**				
**All States**	2724	0.98	(0.89, 1.07)	0.619
**Stratified by College-aged Cohort**	**1960**			
Pre-*Brown* (< 1955)	517	1.03	(0.85, 1.24)	0.784
Post-*Brown*/Pre-CRA (1955 – 1964)	886	0.87	(0.71, 1.06)	0.171
Post-CRA (> 1964)	557	1.46	(0.91, 2.33)	0.117

Abbreviations: REGARDS = REasons for Geographic and Racial Differences in Stroke, HBCU = historically Black college or university, PWI = predominantly White institution, HR = hazard ratio, CI = confidence interval

Estimates were adjusted for sex, community size at birth, college-aged years, mother/female caregiver’s education, and childhood general health, encouragement to succeed in school, death of a parent in childhood, and state of high school attended.

## Data Availability

The data underlying this article were provided by REGARDS under licence / by permission. Data will be shared on request to the corresponding author with permission of REGARDS.
