# Supplementary material for: Attending an Historically Black College or University and all-cause mortality in US Black adults
Source: Am J Epidemiol. Author manuscript; Available in PMC 2026 Aug 17. (PMC13480965; doi:10.1093/aje/kwag141)
Supplement: Supplementary file 1 [file NIHMS2194176-supplement-supplemental.docx]

SUPPLEMENTAL MATERIAL

Title: Attending an Historically Black College or University and all-cause mortality in US Black adults

Authors: Marilyn D. Thomas^1-3^, Carol Wei^2^, Min Hee Kim^4^, Jennifer Manly^7^, Suzanne E. Judd^6^, Justin S. White^8^, Virginia J. Howard^9^, Christina Mangurian^2,3,5^, Rita Hamad^10^, M. Maria Glymour^11^

Affiliations:

^1^ Department of Medicine, Division of General Internal Medicine, University of California San Francisco

^2^ Department of Epidemiology and Biostatistics, University of California San Francisco

^3^ Philip R. Lee Institute for Health Policy Studies, University of California San Francisco

^4^ Center for Health Services Research, Institute for Health, Health Care Policy and Aging, School of Nursing, Rutgers University, New Brunswick, NJ.

^5^ Department of Psychiatry and Behavioral Sciences, University of California San Francisco

^6^ Department of Health Behavior, School of Public Health, University of Alabama at Birmingham

^7^ Columbia University Irving Medical Center, New York, NY

^8^ Department of Health Law, Policy, & Management, Boston University School of Public Health

^9^ Department of Epidemiology, School of Public Health, University of Alabama at Birmingham

^10^ Department of Social and Behavioral Sciences, Harvard T.H. Chan School of Public Health

^11^ Department of Epidemiology, Boston University School of Public Health

APPENDICES

Figures S1-S3

Tables S1-S3

Figure S1. Directed acyclic graph representing working knowledge of factors linking attendance at an Historically Black College or University (HBCU) and later-life cognition, which included demographics and childhood academic support, social support, socioeconomic status (SES), health, and state level-effects. High school proximity to an HBCU was identified as having a direct influence on HBCU attendance.

Figure S2. Kaplan Meier survival curves among Black adults in the REasons for Geographic and Racial Differences in Stroke (REGARDS) study comparing those who attended an historically Black college or university (HBCU) or a predominantly White institution (PWI). The top graph shows main findings among participants who attended high school in a state with an HBCU. The bottom graph shows secondary findings among participants in all states.

```
Test of proportional-hazards assumption

	Time function: Analysis time
	--------------------------------------------------------
			 |        rho     chi2       df 	Prob>chi2
	-------------+------------------------------------------
	 hbcu_status |   -0.03638     0.97        1       0.3242
   age |    0.00529     0.04        1       0.8333
		gender |    0.05011     1.89        1       0.1688
	college-aged |   -0.02685     1.01        1       0.3143
	   comm size |    0.00755     0.05        1       0.8184
 mother educ |   -0.02197     0.66        1       0.4159
	child health |    0.00299     0.01        1       0.9425
	encouragement|    0.02749     0.41        1       0.5210
	parent death |   -0.00062     0.00        1       0.9899
	-------------+------------------------------------------
	 Global test |               10.19        9       0.3356
	--------------------------------------------------------
	Note: Robust variance–covariance matrix used.
```

Figure S3. Assessment of the proportional hazards assumption. The top graph presents the predicted and observed survival estimates among Black adults in the REasons for Geographic and Racial Differences in Stroke (REGARDS) study comparing those who attended a predominantly White institution (left graph) and an historically Black college or university (HBCU, right graph). Each graph shows fully adjusted estimates among participants who attended high school in a state with an HBCU. The bottom table reports the detailed and global test based on Schoenfeld residuals after fitting the Cox model.

| Table S1. Associations between covariates within each childhood domain | | |
| --- | --- | --- |
| **Associations by Covariate Domain** | **chi-square value** | **p-value** |
| **Socioeconomic Status** | | |
| male and female caregivers' education | 1100.000 | <0.001 |
| female caregivers' education and house type | 37.554 | <0.001 |
| female caregivers' education and home ownership | 238.645 | <0.001 |
| female caregivers' education and land ownership | 95.349 | <0.001 |
| male caregivers' education and house type | 31.001 | <0.001 |
| male caregivers' education and home ownership | 155.545 | <0.001 |
| male caregivers' education and land ownership | 40.963 | <0.001 |
| **Academics** | | |
| homework help and encouragement to succeed | 1900.000 | <0.001 |
| **Health** | | |
| general health and birth weight | 31.969 | <0.001 |
| general health and days of hunger | 100.547 | <0.001 |
| birth weight and days of hunger | 0.447 | 0.978 |
| **Social Support** | | |
| parent in jail and death of a parent | 13.627 | <0.001 |
| parent in jail and witnessed domestic violence | 349.202 | <0.001 |
| shown love/affection and witnessed domestic violence | 247.689 | <0.001 |
| shown love/affection and death of parent | 25.407 | <0.001 |
| shown love/affection and parent in jail | 61.771 | <0.001 |

| Table S2. Bivariable associations of covariates with mortality and HBCU status | | | | | | |
| --- | --- | --- | --- | --- | --- | --- |
| **Variable** |  | **Mortality-Covariate Association** | |  | **HBCU-Covariate Association** | |
|  |  | **chi-square value** | **p-value** |  | **chi-square value** | **p-value** |
| **Socioeconomic Status** | | | |  |  |  |
| dad/male caregiver education |  | 5.314 | 0.150 |  | 21.360 | <0.001 |
| **mom/female caregiver education** |  | **13.125** | **<0.001** |  | **46.749** | **<0.001** |
| type of housing |  | 1.835 | 0.399 |  | 20.571 | <0.001 |
| land ownership |  | 3.154 | 0.076 |  | 15.789 | <0.001 |
| home ownership |  | 9.323 | 0.009 |  | 34.558 | <0.001 |
| **Academics** | | | |  |  | |
| homework help |  | 1.737 | 0.420 |  | 33.439 | <0.001 |
| **encouragement to succeed in school** |  | **1.701** | **0.791** |  | **75.124** | **<0.001** |
| **Health** | | | |  |  | |
| **general health** |  | **1.949** | **0.163** |  | **1.703** | **0.192** |
| birth weight |  | 0.184 | 0.912 |  | 0.194 | 0.907 |
| days of hunger |  | 1.404 | 0.496 |  | 10.352 | 0.006 |
| **Social Support** | | | |  |  | |
| shown love and affection |  | 1.205 | 0.547 |  | 15.039 | 0.079 |
| **parental death** |  | **12.788** | **< 0.001** |  | **3.085** | **<0.001** |
| witnessed domestic violence |  | 3.769 | 0.052 |  | 15.402 | < 0.001 |
| **State-level** |  | **T-statistic** | **p-value** |  | **T-statistic** | **p-value** |
| Black population |  | 2.535 | 0.012 |  | -12.124 | < 0.001 |
| Hispanic population |  | -1.850 | 0.065 |  | 12.703 | < 0.001 |
| foreign born |  | -2.663 | 0.008 |  | 16.626 | < 0.001 |
| **below federal poverty** |  | **2.870** | **0.004** |  | **-13.095** | **< 0.001** |
| Abbreviations: HBCU = historically Black College or University | | | | | | |
| Note: Adjusted covariates in bold. | | | | | | |

| Table S3. Characteristics of 1,960 REGARDS participants who attended high school in a state with an HBCU by HBCU status and years when college-aged | | | | | | | | | | | | | |
| --- | --- | --- | --- | --- | --- | --- | --- | --- | --- | --- | --- | --- | --- |
|  |  | **< 1955** | | | | **1955-64** | | | | **> 1964** | | | |
| Variables |  | (n=205) | (n=312) | (N=517) |  | (n=318) | (n=568) | (N=886) |  | (n=171) | (n=386) | (N=557) |  |
|  |  | HBCU | PWI | Total |  | HBCU | PWI | Total |  | HBCU | PWI | Total |  |
|  |  | *n (%)* | | | *p-value* | *n (%)* | | | *p-value* | *n (%)* | | | *p-value* |
| **Baseline age** (mean, SD) |  | 72.5 (5.1) | 72.4 (4.5) | 72.5 (4.8) | 0.938 | 61.4 (2.9) | 61.2 (3.2) | 61.3 (3.1) | 0.438 | 53.4 (3.4) | 52.5 (3.6) | 52.8 (3.6) | 0.004 |
| **Sex** |  |  |  |  |  |  |  |  |  |  |  |  |  |
| Male |  | 80 (39.0) | 113 (36.2) | 193 (37.3) | 0.519 | 113 (35.5) | 204 (35.9) | 317 (35.8) | 0.910 | 38 (22.2) | 94 (24.4) | 132 (23.7) | 0.586 |
| Female |  | 125 (61.0) | 199 (63.8) | 324 (62.7) |  | 205 (64.5) | 364 (64.1) | 569 (64.2) |  | 133 (77.8) | 292 (75.6) | 425 (76.3) |  |
| **Community size at birth** |  |  |  |  |  |  |  |  |  |  |  |  |  |
| City/Large Town |  | 59 (28.8) | 97 (31.1) | 156 (30.2) | 0.847 | 125 (39.3) | 203 (35.7) | 328 (37.0) | 0.359 | 62 (36.3) | 145 (37.5) | 207 (37.1) | 0.882 |
| Rural/Small Town |  | 139 (67.8) | 204 (65.4) | 343 (66.3) |  | 179 (56.3) | 346 (60.9) | 525 (59.3) |  | 103 (60.2) | 225 (58.3) | 328 (58.9) |  |
| Missing |  | 7 (3.4) | 11 (3.5) | 18 (3.5) |  | 14 (4.4) | 19 (3.4) | 33 (3.7) |  | 6 (3.5) | 16 (4.2) | 22 (4.0) |  |
| **Mother's/Caregiver's education** |  |  |  |  |  |  |  |  |  |  |  |  |  |
| College |  | 50 (24.4) | 24 (7.7) | 74 (14.3) | < 0.001 | 57 (17.9) | 43 (7.6) | 100 (11.3) | < 0.001 | 30 (17.5) | 41 (10.6) | 71 (12.8) | 0.106 |
| Post HS Vocational/Professional |  | 4 (1.9) | 17 (5.5) | 21 (4.1) |  | 26 (8.2) | 29 (5.1) | 55 (6.2) |  | 14 (8.2) | 28 (7.3) | 42 (7.5) |  |
| HS/GED |  | 19 (9.3) | 25 (8.0) | 44 (8.5) |  | 44 (13.9) | 83 (14.6) | 127 (14.3) |  | 37 (21.6) | 77 (19.9) | 114 (20.5) |  |
| <HS/None/Unknown |  | 128 (62.4) | 241 (77.2) | 369 (71.4) |  | 188 (59.1) | 402 (70.8) | 590 (66.6) |  | 90 (52.6) | 237 (61.4) | 327 (58.7) |  |
| Missing |  | 4 (2.0) | 5 (1.6) | 9 (1.7) |  | 3 (0.9) | 11 (1.9) | 14 (1.6) |  | 0 (0) | 3 (0.8) | 3 (0.5) |  |
| **Childhood encouragement to succeed in school** |  |  |  |  |  |  |  |  |  |  |  |  |  |
| All the time |  | 149 (72.7) | 187 (60.0) | 336 (65.0) | 0.030 | 230 (72.3) | 315 (55.4) | 545 (61.5) | < 0.001 | 125 (73.1) | 216 (56.0) | 341 (61.2) | 0.006 |
| Most of the time |  | 24 (11.7) | 49 (15.7) | 73 (14.1) |  | 45 (14.2) | 94 (16.6) | 139 (15.7) |  | 21 (12.3) | 61 (15.8) | 82 (14.7) |  |
| Some of the time |  | 13 (6.3) | 27 (8.7) | 40 (7.7) |  | 11 (3.5) | 59 (10.4) | 70 (7.9) |  | 11 (6.4) | 44 (11.4) | 55 (9.9) |  |
| Little of time |  | 4 (2.0) | 16 (5.1) | 20 (3.9) |  | 10 (3.1) | 46 (8.1) | 56 (6.3) |  | 7 (4.1) | 28 (7.2) | 35 (6.3) |  |
| None of the time |  | 2 (1.0) | 12 (3.8) | 14 (2.7) |  | 5 (1.5) | 33 (5.8) | 38 (4.3) |  | 3 (1.8) | 22 (5.7) | 25 (4.5) |  |
| Missing |  | 13 (6.3) | 21 (6.7) | 34 (6.6) |  | 17 (5.4) | 21 (3.7) | 38 (4.3) |  | 4 (2.3) | 15 (3.9) | 19 (3.4) |  |
| **Childhood general health** |  |  |  |  |  |  |  |  |  |  |  |  |  |
| Excellent/Very Good |  | 136 (66.3) | 196 (62.8) | 332 (64.2) | 0.716 | 228 (71.7) | 393 (69.2) | 621 (70.1) | 0.137 | 123 (71.9) | 292 (70.5) | 395 (70.9) | 0.892 |
| Good/Fair/Poor |  | 63 (30.7) | 106 (34.0) | 169 (32.7) |  | 79 (24.8) | 165 (29.0) | 244 (27.5) |  | 43 (23.1) | 104 (26.9) | 147 (26.4) |  |
| Missing |  | 6 (3.0) | 10 (3.2) | 16 (3.1) |  | 11 (3.5) | 10 (1.8) | 21 (2.4) |  | 5 (3.0) | 10 (2.6) | 15 (2.7) |  |
| **Death of a parent in childhood** |  |  |  |  |  |  |  |  |  |  |  |  |  |
| No/Unknown |  | 116 (56.6) | 163 (52.2) | 279 (54.0) | 0.013 | 219 (68.9) | 328 (57.8) | 547 (61.7) | 0.005 | 117 (68.4) | 232 (60.1) | 349 (62.7) | 0.173 |
| Yes |  | 63 (30.7) | 78 (25.0) | 141 (27.3) |  | 62 (19.5) | 146 (25.7) | 208 (23.5) |  | 35 (20.5) | 99 (25.6) | 134 (24.0) |  |
| Missing |  | 26 (12.7) | 71 (22.8) | 97 (18.7) |  | 37 (11.6) | 94 (16.5) | 131 (14.8) |  | 19 (11.1) | 55 (14.3) | 74 (13.3) |  |
| **Follow-up interviews** (mean, SD) |  | 7.7 (3.7) | 7.6 (3.5) | 7.6 (3.6) | 0.749 | 9.3 (3.4) | 9.2 (3.5) | 9.2 (3.5) | 0.800 | 9.5 (3.5) | 9.8 (3.4) | 9.7 (3.4) | 0.442 |
| **Survival to 2023** |  |  |  |  |  |  |  |  |  |  |  |  |  |
| No |  | 96 (46.8) | 147 (47.1) | 243 (47.0) | 0.949 | 260 (81.8) | 449 (79.1) | 709 (80.0) | 0.333 | 145 (84.8) | 352 (91.2) | 497 (89.2) | 0.025 |
| Yes |  | 109 (53.2) | 165 (52.9) | 274 (53.0) |  | 58 (18.2) | 119 (20.9) | 177 (20.0) |  | 26 (15.2) | 34 (8.8) | 60 (10.8) |  |
| **Age at death** (mean, SD) |  | 88.5 (5.5) | 88.3 (5.1) | 88.4 (5.2) | 0.654 | 75.7 (4.2) | 76.7 (4.2) | 76.4 (4.2) | 0.152 | 68.1 (5.2) | 66.4 (5.2) | 67.1 (4.8) | 0.172 |
| Abbreviations: REGARDS = REasons for Geographic and Racial Differences in Stroke, PWI = predominantly White institution, HBCU = historically Black college or university, HS = high school, GED = general education diploma, SD = standard deviation | | | | | | | | | | | | | |
